# Midregional pro atrial natriuretic peptide: a novel important biomarker for noise annoyance-induced cardiovascular morbidity and mortality?

**DOI:** 10.1007/s00392-020-01645-6

**Published:** 2020-04-18

**Authors:** Omar Hahad, Philipp S. Wild, Jürgen H. Prochaska, Andreas Schulz, Karl J. Lackner, Norbert Pfeiffer, Irene Schmidtmann, Matthias Michal, Manfred Beutel, Andreas Daiber, Thomas Münzel

**Affiliations:** 1 Center for Cardiology-Cardiology I, University Medical Center of the Johannes Gutenberg-University Mainz, Langenbeckstraße 1, 55131 Mainz, Germany; 2 German Center for Cardiovascular Research (DZHK), Partner site Rhine-Main, Mainz, Germany; 3 Preventive Cardiology and Preventive Medicine, Center for Cardiology, University Medical Center of the Johannes Gutenberg-University Mainz, Mainz, Germany; 4 Center for Thrombosis and Hemostasis, University Medical Center of the Johannes Gutenberg-University Mainz, Mainz, Germany; 5 Institute of Clinical Chemistry and Laboratory Medicine, University Medical Center of the Johannes Gutenberg-University Mainz, Mainz, Germany; 6 Department of Ophthalmology, University Medical Center of the Johannes Gutenberg-University Mainz, Mainz, Germany; 7 Institute of Medical Biostatistics, Epidemiology and Informatics, University Medical Center of the Johannes Gutenberg-University Mainz, Mainz, Germany; 8 Department of Psychosomatic Medicine and Psychotherapy, University Medical Center of the Johannes Gutenberg-University Mainz, Mainz, Germany

**Keywords:** Noise annoyance, Risk factor, Midregional pro atrial natriuretic peptide, Cardiovascular disease, Mortality

## Abstract

**Background:**

Environmental noise exposure has been associated with increased cardiovascular morbidity and mortality. Recently, noise annoyance was shown to induce atrial fibrillation, which was accompanied by significantly increased levels of midregional pro atrial natriuretic peptide (MR-proANP). Therefore, the aim of the present study was to analyze the association between noise annoyance, MR-proANP, incident cardiovascular events, and all-cause mortality.

**Methods:**

Levels of MR-proANP were measured in the first 5000 participants of the population-based Gutenberg Health Study. Annoyance was assessed separately for aircraft, road traffic, railway, neighborhood, and industrial/construction noise during the day and sleep.

**Results:**

In cross-sectional analyses, aircraft noise annoyance during day and sleep, industrial/construction noise annoyance during day, and railway noise annoyance during sleep were independently associated with increased levels of MR-proANP after multivariable adjustment. After a 5-year follow-up period, there were 43 cases of incident atrial fibrillation and 103 of incident cardiovascular disease (comprising atrial fibrillation, coronary artery disease, myocardial infarction, heart failure, or stroke). Moreover, there were 301 deaths after a mean follow-up of 7.42 ± 1.66 years. An odds ratio (OR) of 2.82 ([95% confidence interval (CI) 1.86; 4.35], *p* < 0.0001) for incident atrial fibrillation and an OR of 1.49 ([95% CI 1.13; 1.96], *p* = 0.0046) for incident cardiovascular disease per 1-standard deviation (SD) increase in MR-proANP levels were found. A 36% (hazard ratio: 1.36 [95% CI 1.19; 1.55], *p* < 0.0001) higher risk of death was found per 1-SD increase in MR-proANP levels.

**Conclusions:**

Noise annoyance may contribute to cardiovascular morbidity and mortality and is characterized by increased levels of MR-proANP.

**Graphic abstract:**

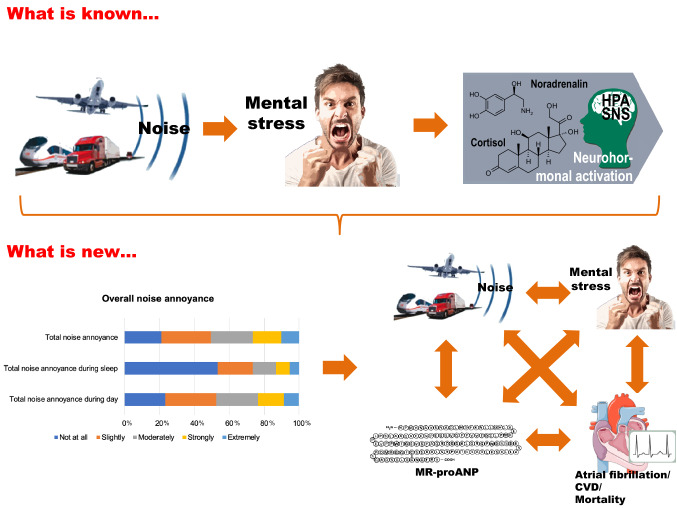

## Introduction

Environmental noise exposure is a well-established risk factor for cardiovascular disease [1]. Over the past decade, several high-quality studies support that traffic noise exposure, the most significant source of noise pollution in Europe, is associated with increased risk of arterial hypertension, ischemic heart disease, stroke, and myocardial infarction [2, 3]. Babisch’s noise reaction model and the non-auditory pathway are regarded as the central framework by which noise induces adverse health effects [4, 5]. In this context, noise can negatively affect communication, daily activities, and sleep, leading to annoyance, mental stress, and subsequent sympathetic and endocrine activation [2, 6, 7]. If the exposure becomes chronic, noise initiates a pathophysiological cascade, resulting in increased stress hormone levels, blood pressure, and heart rate, which in turn promotes the manifestation of cardiovascular risk factors and diseases [8]. However, mechanistic insight by which noise triggers detrimental effects on the cardiovascular system is still elusive. Recent human studies suggest that traffic noise exposure causes the release of stress hormones and inflammatory signaling molecules leading to oxidative stress and subsequent endothelial dysfunction and arterial stiffening [9–12]. We have also demonstrated that noise annoyance is associated with a higher risk of prevalent atrial fibrillation, which was further accompanied by an increased trend of midregional pro atrial natriuretic peptide (MR-proANP) plasma levels [13]. Midregional pro atrial natriuretic peptide mirrors vascular (endothelial) activation and has been correlated with endothelial dysfunction in humans including flow-mediated vasodilation and markers of peripheral arterial tonometry [14]. Furthermore, assessment of MR-proANP has been shown to be useful in patients with prevalent cardiovascular disease as an indicator of cardiovascular risk and mortality in initially healthy subjects [15]. Therefore, the present study aimed to investigate the relationship between noise annoyance, levels of MR-proANP, incident cardiovascular events, and mortality in the large population-based Gutenberg Health Study (GHS).

## Methods

### Study design and sample

The analysis was based on data from the GHS, a population-based, prospective, observational single-center cohort study from Mid-Western Germany [16]. The sample was drawn randomly from the local registry in the city of Mainz and the district of Mainz-Bingen. Insufficient knowledge of German language, psychological, or physical impairment hindering participation in the study led to exclusion. The GHS puts main emphasis on cardiovascular risk stratification, as well as focus on metabolic, ophthalmological, cancer, immune system, and mental diseases. The age-, sex-, and residence-stratified study sample included 15,010 individuals (age range 35–74 years) of the baseline examination performed from April 2007 to April 2012 at the University Medical Center Mainz, Germany. Assessment of MR-proANP was conducted in the first 5000 individuals, thus analyses were restricted to this subpopulation. In a standardized 5-h-long baseline-examination, individuals underwent a variety of interviews and clinical examinations with comprehensive assessment of lifestyle, psychosocial, environmental, and laboratory parameters according to standard operating procedures. Follow-up examinations were performed 5 years after enrollment, i.e. from 2012 to 2017. The GHS and its study procedures were approved by the ethics committee of the Statutory Physician Board of the State Rhineland-Palatinate [Reference No. 837.020.07(5555)] and the local data safety commissioners. The study design was in line with the tenets of the revised Helsinki protocol and principles outlined in recommendations for Good Clinical and Epidemiological Practice. Written informed consent was obtained from all individuals prior to participation.

### Noise annoyance

Noise annoyance was assessed in accordance to previous protocols published by Felscher-Suhr, Guski and Schluemer [17]. Participants were asked to rate “how annoyed have you been in the past years by…” using a 5-point Likert scale ranging from “not at all”, over “slightly”, “moderately”, and “strongly” to “extremely”. Multiple sources of annoyance including road traffic, aircraft, railway, industrial/construction, and neighborhood noise “during the day” and “in your sleep” were assessed.

### Measures

Venous blood was obtained after an overnight fast (at least 8 h) and samples were processed for plasma and stored in aliquots at − 80 °C immediately after blood draw. Routine laboratory methods were used for blood glucose and lipid measurements. In brief, MR-proANP was determined using a commercial available assay (immunoluminometric assay MR-proANP, Brahms GmbH, Hennigsdorf/Berlin, Germany) according to the manufacturer’s instructions as reported previously [14]. Reproducibility was good, with all of the coefficients of variation (intra-assay and inter-assay) < 5%. Medication history was derived from medical records and was categorized according to the Anatomical Therapeutic Chemical Classification System [18].

Further variables were defined as follows: arterial hypertension was defined as systolic blood pressure ≥ 140 mmHg or diastolic blood pressure ≥ 90 mmHg at rest (mean of 2nd and 3rd standardized measurement after 8 and 11 min of rest), or by intake of any antihypertensive drugs within the last 2 weeks, or arterial hypertension diagnosed by a physician. Diabetes mellitus was defined by already diagnosed or a fasting blood glucose level (overnight fast of at least 8 h) ≥ 126 mg/dL or HbA1c > 6.5%, or a non-fasting blood glucose level (less than 8 h of fasting) ≥ 200 mg/dL. Also, subjects with intake of oral blood glucose-lowering drugs or receiving insulin therapy were classified as diabetic. Dyslipidemia was defined as at least one of the following: physician diagnosis of dyslipidemia, low-density lipoprotein cholesterol/high-density lipoprotein cholesterol ratio > 3.5, or triglycerides ≥ 150 mg/dL. A positive family history of myocardial infarction or stroke was recorded in a female first-degree relative ≤ 65 years or in a male first-degree relative ≤ 60 years. Never and former smokers were defined as non-smokers and current smokers as smokers. Body mass index was used for the definition of obesity following the World Health Organization (WHO) criteria [19]. Family history of myocardial infarction or stroke was self-reported. Incident cardiovascular disease was assessed on the basis of medical history or diagnosis during a standardized interview as any of the following: atrial fibrillation, coronary artery disease, myocardial infarction, heart failure, or stroke.

Mortality updates were performed by quarterly queries to the registry offices and the mortality registry Rhineland-Palatinate. For death reviews, official death certificates were acquired. Socioeconomic status was assessed by a validated index score comprising information about educational background, current occupation, and income (ranging from 3 to 21) with a higher score indicating higher socioeconomic status [20]. Night shift work was considered as current or non-current. Further details on measurements have been reported previously [21, 22].

### Statistical analysis

Baseline characteristics are shown according to total noise annoyance as absolute and relative frequency for categorical variables and as mean value and standard deviation (SD) or median with 25th and 75th percentiles for continuous variables. Statistical comparisons for categorical variables were made by Fisher exact or *χ*^2^ tests and for continuous variables Mann–Whitney *U* or Student *t* tests were used, respectively. Total noise annoyance was defined as highest annoyance rating regardless of the specific noise source and of whether it affected daytime or sleep. Linear regression analysis with corresponding beta estimates (*β*) were used to assess the association between noise annoyance and levels of MR-proANP. Odds ratios (OR) from a logistic regression model were used to analyze the impact of MR-proANP on incident cardiovascular events. For analyses of all-cause mortality, Cox proportional hazard regression models with corresponding hazard ratios (HR) were used. All regression models were adjusted sequentially. Because of the explorative nature of the study, no Bonferroni correction of *p* values was conducted. *p* values should be treated as a continuous measure of statistical strength of an association and they are therefore reported exactly. All tests were two-sided and *p* values < 0.05 were considered significant. The statistical data analyses were performed using the software R, version 3.5.1 (https://www.r-project.org/).

## Results

### Baseline characteristics

A total of 4826 subjects answered the questions about noise annoyance. Baseline *characteristics* of this study sample stratified by the degree of total noise annoyance are displayed in Table 1. Nearly 80% of the subjects reported being affected by noise annoyance to a certain degree (not at all: 21.1%, slightly: 28.4%, moderately: 24.0%, strongly: 16.2%, extremely: 10.3%). Extremely annoyed subjects were more likely to be women (54.2%), had the highest prevalence of current smoking (23.0%), and highest use of antihypertensives (1.6%) and diuretics (6.7%). Midregional pro atrial natriuretic peptide levels tended to increase from no to extreme annoyance (65.7 to 69.0 pmol/L). No clear trend was observed in the case of other sociodemographic variables, cardiovascular risk factors, and medication use.

**Table 1:** Baseline characteristics of the study sample by degree total noise annoyance (*n* = 4826)

Variable	Not at all (*n* = 1018)	Slightly (*n* = 1372)	Moderately (*n* = 1160)	Strongly (*n* = 780)	Extremely (*n* = 496)	*p* value
Female sex, no. (%)	529 (52.0)	631 (46.0)	561 (48.4)	378 (48.5)	269 (54.2)	**0.016**
Age, years	56.8 ± 10.7	54.2 ± 10.7	55.6 ± 11.2	54.5 ± 11.1	55.5 ± 10.8	0.65
Socioeconomic status	11.99 ± 4.38	13.19 ± 4.42	12.67 ± 4.34	12.90 ± 4.49	12.40 ± 4.41	0.13
Time at current residence, years	20.65 ± 15.40	19.26 ± 14.65	20.80 ± 15.44	19.80 ± 14.92	19.46 ± 15.21	0.38
Night shift work, no. (%)	251 (24.7)	349 (25.4)	301 (25.9)	233 (29.9)	131 (26.4)	0.91
Cardiovascular risk factors						
MR-proANP (pmol/L)	65.7 (49.9/89.5)	63.6 (47.7/85.4)	67.2 (50.0/89.8)	65.0 (48.0/90.9)	69.0 (51.0/96.0)	**0.013**
Diabetes mellitus, no. (%)	119 (11.7)	110 (8.1)	109 (9.5)	77 (9.9)	45 (9.1)	0.81
Hypertension, no. (%)	534 (52.5)	684 (49.9)	605 (52.2)	382 (49.0)	246 (49.6)	0.60
Current smoking, no. (%)	205 (20.1)	247 (18.0)	227 (19.6)	141 (18.1)	114 (23.0)	**0.036**
Obesity, no. (%)	292 (28.7)	293 (21.4)	273 (23.5)	182 (23.3)	108 (21.8)	0.29
Dyslipidemia, no. (%)	479 (47.1)	581 (42.3)	527 (45.5)	339 (43.6)	230 (46.5)	0.42
Family history of myocardial infarction or stroke, no. (%)	242 (23.8)	315 (23.0)	270 (23.3)	170 (21.8)	118 (23.8)	0.69
Medication, no. (%)						
Antidiabetic medication (A10)	71 (7.0)	63 (4.6)	68 (5.9)	53 (6.8)	31 (6.3)	0.76
Antithrombotic agents (B01)	139 (13.7)	137 (10.0)	163 (14.1)	91 (11.7)	64 (13.0)	0.66
Antihypertensives (C02)	13 (1.3)	11 (0.8)	7 (0.6)	9 (1.2)	8 (1.6)	0.15
Diuretics (C03)	64 (6.3)	57 (4.2)	73 (6.3)	49 (6.3)	33 (6.7)	0.36
Beta-blockers (C07)	174 (17.1)	216 (15.8)	196 (16.9)	113 (14.5)	78 (15.8)	0.90
Calcium channel blocker (C08)	86 (8.5)	80 (5.9)	80 (6.9)	58 (7.4)	40 (8.1)	0.41
Agents acting on the renin–angiotensin–aldosterone system (C09)	249 (24.5)	278 (20.3)	276 (23.8)	175 (22.5)	116 (23.5)	0.69
Lipid modifying agents (C10)	160 (15.8)	153 (11.2)	150 (12.9)	103 (13.2)	66 (13.4)	0.89

Statistically significant *p* values (i.e. < 0.05) are given in bold font

Plus–minus values are means ± standard deviations and two values in parentheses are medians with 25th and 75th percentiles. *P* value for the comparison of extreme annoyance vs rest. Medication is labeled with the anatomical therapeutic chemical-code

### Noise annoyance

**Fig. 1:**
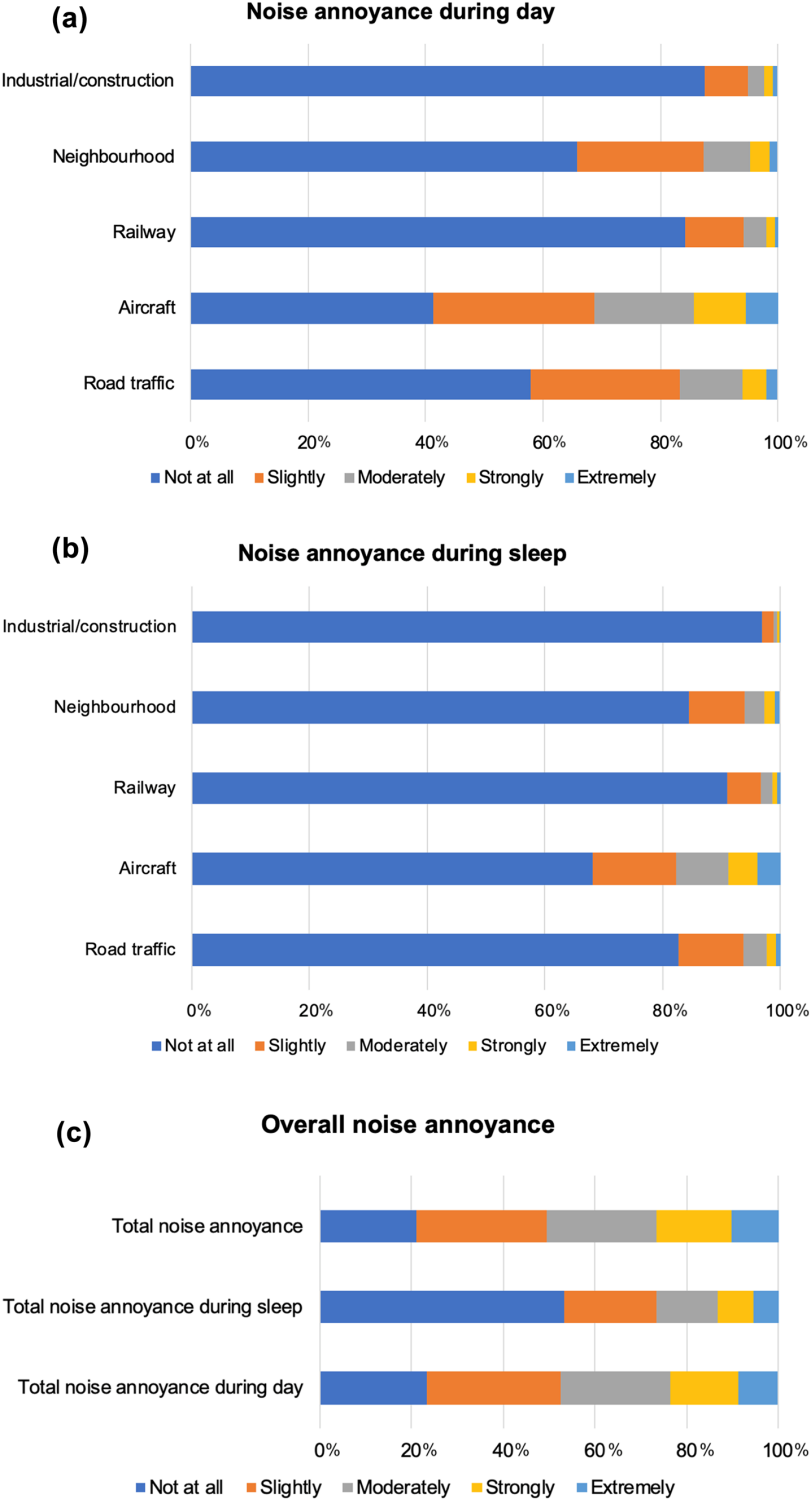
Degrees of noise annoyance during (**a**) day and (**b**) sleep according to different sources of noise and (**c**) overall

As evident in Fig. 1, aircraft noise was the leading source of annoyance in the population affecting nearly 60% during day and more than 30% during sleep. Moreover, aircraft noise was the leading source of strong and extreme annoyance (day: 14.4%, sleep: 8.9%). This was followed by road traffic (day: 41.1%, sleep: 17.2%), neighborhood (day: 34.2%, sleep: 15.6%), railway (day: 15.7%, sleep: 9.0%), and industrial/construction (day: 12.5%, sleep: 3.1%) noise annoyance. Overall, total noise annoyance was higher during the day than sleep, affecting 76.6% during the day and 46.8% during sleep.

### Association between noise annoyance and MR-proANP

Aircraft noise annoyance during day (β: 0.016 [95% CI 0.0070; 0.025], *p* = 0.00049 per point increase in annoyance) as well as during sleep (β: 0.020 [95% CI 0.010; 0.030], *p* < 0.0001) was independently associated with increased levels of MR-proANP after multivariable adjustment for sex, age, socioeconomic status, length of time at current residence, night shift work, diabetes mellitus, arterial hypertension, current smoking, obesity, dyslipidemia, family history of myocardial infarction or stroke, and medication use (Table 2). Moreover Industrial/construction noise annoyance during day (*β*: 0.019 [95% CI 0.0021; 0.036], *p* < 0.028) and railway noise annoyance during sleep (*β*: 0.028 [95% CI 0.0075; 0.049], *p* = 0.0078) were independently associated with increased MR-proANP levels. Furthermore, total noise annoyance (*β*: 0.013 [95% CI 0.0041; 0.021], *p* = 0.0036) as well as total noise annoyance during day (*β*: 0.014 [95% CI 0.0057; 0.023], *p* = 0.0012) and sleep (*β*: 0.011 [95% CI 0.0019; 0.020], *p* = 0.017) were independently associated with higher levels of MR-proANP.

**Table 2:** Cross-sectional association between noise annoyance and levels of MR-proANP

Noise annoyance	*n*	Model 1		Model 2		Model 3	
		Beta estimate per point increase [95% CI]	*p* value	Beta estimate per point increase [95% CI]	*P* value	Beta estimate per point increase [95% CI]	*p* value
During day							
Road traffic	4,766	0.0037 [− 0.0085; 0.016]	0.55	0.0051 [− 0.0072; 0.017]	0.41	0.0053 [− 0.0061; 0.017]	0.36
Aircraft	4,764	0.017 [0.0077; 0.027]	**0.00046**	0.016 [0.0061; 0.026]	**0.0015**	0.016 [0.0070; 0.025]	**0.00049**
Railway	4,761	0.016 [− 0.0019; 0.034]	0.080	0.018 [− 0.000049; 0.036]	0.051	0.013 [− 0.0035; 0.029]	0.12
Neighborhood	4,764	0.013 [− 0.000043; 0.027]	0.051	0.016 [0.0021; 0.029]	**0.024**	0.0098 [− 0.0027; 0.022]	0.12
Industrial/construction	4,763	0.015 [− 0.0034; 0.034]	0.11	0.016 [− 0.0025; 0.034]	0.091	0.019 [0.0021; 0.036]	**0.028**
During sleep							
Road traffic	4,748	0.0086 [− 0.0086; 0.026]	0.33	0.0100 [− 0.0072; 0.027]	0.26	0.0081 [− 0.0078; 0.024]	0.32
Aircraft	4,746	0.023 [0.012; 0.033]	** < 0.0001**	0.021 [0.0098; 0.031]	**0.00018**	0.020 [0.010; 0.030]	** < 0.0001**
Railway	4,746	0.032 [0.0097; 0.055]	**0.0051**	0.033 [0.010; 0.055]	**0.0041**	0.028 [0.0075; 0.049]	**0.0078**
Neighborhood	4,747	− 0.0014 [− 0.019; 0.016]	0.87	0.0012 [− 0.016; 0.018]	0.89	0.0014 [− 0.015; 0.018]	0.87
Industrial/construction	4,744	0.018 [− 0.019; 0.056]	0.34	0.019 [− 0.019; 0.056]	0.33	0.020 [− 0.014; 0.054]	0.25
Overall							
Total noise annoyance during day	4,766	0.015 [0.0060; 0.025]	**0.0014**	0.015 [0.0059; 0.025]	**0.0014**	0.014 [0.0057; 0.023]	**0.0012**
Total noise annoyance during sleep	4,750	0.012 [0.0021; 0.021]	**0.017**	0.011 [0.0011; 0.020]	**0.029**	0.011 [0.0019; 0.020]	**0.017**
Total noise annoyance	4,767	0.014 [0.0044; 0.023]	**0.0037**	0.013 [0.0043; 0.023]	**0.0041**	0.013 [0.0041; 0.021]	**0.0036**

Statistically significant *p* values (i.e. < 0.05) are given in bold font

Beta estimates and 95% confidence intervals are derived from a linear regression model modeling for MR-proANP levels (dependent variable) per point increase in noise annoyance (independent variable). *n* denotes model 3

Model 1 was adjusted for sex (categorical) and age (continuous)

Model 2 was additionally adjusted for socioeconomic status (continuous), length of time at current residence (continuous), and night shift work (categorical)

Model 3 was additionally adjusted for diabetes mellitus, arterial hypertension, smoking, obesity, dyslipidemia, family history of myocardial infarction or stroke, and medication use (diabetic drugs, antithrombotic agents, antihypertensives, diuretics, beta-blockers, calcium channel blocker, agents acting on the renin–angiotensin–aldosterone system, and lipid modifying agents) (all categorical)

### Impact of MR-proANP on incident cardiovascular events and all-cause mortality

In the next step, impact of MR-proANP on incident atrial fibrillation as well as on incident cardiovascular disease (composite variable comprising incident atrial fibrillation, coronary artery disease, myocardial infarction, heart failure, or stroke; Table 3) and all-cause mortality (Table 4) was evaluated. During the 5-year follow-up period, incident atrial fibrillation and incident cardiovascular disease occurred in *n* = 43 and *n* = 103 subjects, respectively. There were 301 deaths after a mean follow-up of 7.42 ± 1.66 years. After multivariable adjustment, an OR of 2.82 ([95% CI 1.86; 4.35], *p* < 0.0001) for incident atrial fibrillation and an OR of 1.49 ([95% CI 1.13; 1.96], *p* = 0.0046) for incident cardiovascular disease per 1-SD increase in MR-proANP levels were found. In addition, a 36% (HR 1.36 [95% CI 1.19; 1.55], *p* < 0.0001) higher risk of death was found per 1-SD increase in MR-proANP levels.

**Table 3:** Association between levels of MR-proANP and incident atrial fibrillation or cardiovascular disease

	*n*	Model 1		Model 2		Model 3	
		Odds ratio per SD [95% CI]	*p* value	Odds ratio per SD [95% CI]	*P* value	Odds ratio per SD [95% CI]	*p* value
Estimates for incident atrial fibrillation							
MR-proANP	3386	3.31 [2.31; 4.81]	** < 0.0001**	3.19 [2.22; 4.65]	** < 0.0001**	2.82 [1.86; 4.35]	** < 0.0001**
Estimates for incident cardiovascular disease (comprising atrial fibrillation, coronary artery disease, myocardial infarction, heart failure, or stroke)							
MR-proANP	3015	1.33 [1.04; 1.71]	**0.025**	1.33 [1.03; 1.71]	**0.026**	1.49 [1.13; 1.96]	**0.0046**

Statistically significant *p* values (i.e. < 0.05) are given in bold font

Odds ratios and 95% confidence intervals are derived from a logistic regression model modeling for incident atrial fibrillation or cardiovascular disease (dependent variables) per 1-standard deviation increase in MR-proANP levels (independent variable). There were 43 incident cases of atrial fibrillation and 103 incident cases of cardiovascular disease at follow-up. *n* denotes model 3

Model 1 was adjusted for sex (categorical) and age (continuous)

Model 2 was additionally adjusted for socioeconomic status (continuous), length of time at current residence (continuous), and night shift work (categorical)

Model 3 was additionally adjusted for diabetes mellitus, arterial hypertension, smoking, obesity, dyslipidemia, family history of myocardial infarction or stroke, and medication use (diabetic drugs, antithrombotic agents, antihypertensives, diuretics, beta-blockers, calcium channel blocker, agents acting on the renin–angiotensin–aldosterone system, and lipid modifying agents) (all categorical)

**Table 4:** Association between levels of MR-proANP and all-cause mortality

	*n*	Model 1		Model 2		Model 3	
		Hazard ratio per SD [95% CI]	*p* value	Hazard ratio per SD [95% CI]	*p* value	Hazard ratio per SD [95% CI]	*p* value
Estimates for all-cause mortality							
MR-proANP	4830	1.41 [1.26; 1.58]	** < 0.0001**	1.42 [1.27; 1.60]	** < 0.0001**	1.36 [1.19; 1.55]	** < 0.0001**

Statistically significant *p* values (i.e. < 0.05) are given in bold font

Hazard ratios and 95% confidence intervals are derived from a Cox proportional hazard regression model modeling for all-cause mortality (dependent variable) per 1-standard deviation increase in MR-proANP levels (independent variable). There were 301 deaths at follow-up. *n* denotes model 3

Model 1 was adjusted for sex (categorical) and age (continuous)

Model 2 was additionally adjusted for socioeconomic status (continuous), length of time at current residence (continuous), and night shift work (categorical)

Model 3 was additionally adjusted for diabetes mellitus, arterial hypertension, smoking, obesity, dyslipidemia, family history of myocardial infarction or stroke, and medication use (diabetic drugs, antithrombotic agents, antihypertensives, diuretics, beta-blockers, calcium channel blocker, agents acting on the renin–angiotensin–aldosterone system, and lipid modifying agents) (all categorical)

## Discussion

To our knowledge, this is the first study that investigated a close association between noise annoyance and levels of MR-proANP, a marker that reflects vascular endothelial activation and predicts future cardiovascular events in the large cohort of the GHS. We established that aircraft noise annoyance during the day and sleep, industrial/construction noise annoyance during the day, and railway noise annoyance during sleep were independently associated with increased levels of MR-proANP even after multivariable adjustment for sociodemographic variables, cardiovascular risk factors, and medication use. Total noise annoyance, as an indicator of overall annoyance regardless of the specific noise source and whether it affected daytime or sleep, was also independently predictive of MR-proANP, as it was the case for total noise annoyance during day and sleep. Moreover, higher levels of MR-proANP were shown to be associated with an increased risk of incident atrial fibrillation and future cardiovascular disease. Likewise, the risk of death was associated with higher MR-proANP levels. Importantly, these associations were only marginally influenced by stepwise adjustment for confounders, implicating that noise annoyance may constitute an independent risk factor via association with MR-proANP. These findings suggest that noise annoyance may contribute to increased morbidity and mortality and is characterized by increased levels of MR-proANP.

### Noise annoyance in the population—the importance of aircraft noise

Traffic noise, which is steadily increasing due to increasing demand for transportation and growing urbanization, is the major source of noise annoyance in Western European countries. The WHO estimates that traffic-related noise exposure is responsible for a yearly loss of up to 1.6 million years of healthy life in Western Europe, mainly due to sleep disturbances and annoyance reactions [23]. Annoyance by chronic low-level noise exposure and its interference with daily activities and sleep can lead to increased stress hormone levels (e.g. cortisol and noradrenaline), blood pressure, and heart rate via activation of the hypothalamus–pituitary–adrenal axis or the sympathetic nervous system, which in turn favors the development and acceleration of cardiovascular risk factors and diseases [2, 7, 24]. Besides this, chronic noise exposure and related annoyance are also associated with mental disorders such as depression and anxiety, cognitive dysfunction, and maladaptive coping mechanisms in the manner of lifestyle risk factors such as physical inactivity, smoking, and alcohol consumption that may contribute to increased risk of cardiovascular disease [8, 25].

In this subsample of the GHS, noise annoyance was a major environmental problem with an epidemic character, affecting almost 80% of the participants. In particular, aircraft noise was the leading source of annoyance, affecting the most participants during day and sleep, which is in line with previous results of the GHS [13, 26]. In the present study, aircraft noise was the only source of annoyance that was associated with increased levels of MR-proANP during the day as well as during sleep, while aircraft noise annoyance during sleep had an even stronger impact on MR-proANP levels than during the day. In general, effect estimates were higher during nighttime than during daytime, which goes along with previous (pre)clinical studies indicating that in particular nighttime aircraft noise induces adverse cardiovascular effects, including increased stress hormone release, elevated blood pressure, endothelial dysfunction, and increased arterial stiffness [9, 10, 27, 28]. This may reflect the adverse effect of nighttime noise on sleep quality and its importance for cardiovascular health [29].

### Midregional pro atrial natriuretic peptide as a biomarker for increased risk in the context of noise annoyance

The natriuretic peptide MR-proANP is a cardiac hormone released by the atrium, which effectively controls blood pressure and plasma volume through inhibition of the renin–angiotensin–aldosterone system and catecholamine release and stimulation of natriuresis and vasodilation in response to wall stretch [30]. Its role for cardiovascular prognosis is well-established, showing that its assessment is useful in several cardiovascular disease phenotypes including chronic and acute heart failure, atrial fibrillation, hypertension, acute myocardial infarction, and stroke [15, 31]. Likewise, MR-proANP has been shown e.g. to improve prediction of mortality in patients with ST-segment elevation myocardial infarction [32] and to represent one of the strongest predictors of cardiovascular outcome in stable angina [33].

Further, MR-proANP has been directly correlated with endothelial dysfunction obtained by various noninvasive vascular function testing methodologies [14, 34]. Endothelial dysfunction is regarded as an early key event in the development of manifest cardiovascular disease with studies indicating its predictive role for the future development of cardiovascular events and mortality risk [35]. Indeed, experimental animal and human studies have shown that traffic noise-induced autonomic perturbation and sympathoadrenal activation lead to increased levels of circulating stress hormones and oxidative stress-induced endothelial dysfunction accompanied by the release of proinflammatory mediators, increased oxidative stress [9, 10, 24, 28], and activation of prothrombotic pathways [12]. Thus, MR-proANP may reflect a compensatory mechanism to reduce the cardiovascular load in response to increased noise stress. Of note, since noise annoyance also reflects mental stress and has been associated with psychological disorders, it is important to mention that growing evidence suggests that MR-proANP play a role in the neurobiology of affective disorders modulating emotional and endocrine stress responses [26, 36]. In addition, recent studies have shown that chronic exposure to transportation noise caused by aircraft and road traffic noise is associated with higher amygdala activity, a limbic structure that is involved in stress perception, leading to vascular inflammation and increased cardiovascular event rates [37, 38]. In support of this, MR-proANP was recently shown to predict impaired physical and mental quality of life [39]. Consistent with the above-mentioned results, MR-proANP was a strong predictor of death as well as incident atrial fibrillation and cardiovascular events further comprising coronary artery disease, myocardial infarction, heart failure, and stroke. Thus, these findings may provide mechanistic insight by which environmental noise induces adverse cardiovascular effects.

### Strengths and limitations

Strengths of the present study include the novelty of examining the association between noise annoyance and levels of MR-proANP in a large population-representative sample. The comprehensive and highly standardized assessment of sociodemographic variables, cardiovascular risk factors, biomarkers, and medication use enabled to adjust for a broad spectrum of confounders. Also, the assessment of various sources of noise annoyance during day and sleep is notable. Some limitations, however, need to be considered. The observational, partly cross-sectional nature of the study does not allow for causal inferences and residual confounding cannot be fully excluded. As no data were available on objective noise levels, we considered annoyance to be a valid indicator of adverse noise-induced effects. This may increase risk of misclassification. Furthermore, we cannot exclude that air pollution (annoyance) may have affected the present results, although studies support the concept that both noise and air pollution independently affect health [40, 41]. Lastly, we cannot evaluate how specific sources of noise annoyance may have interacted or influenced each other.

## Conclusions

Environmental noise-induced annoyance, in particular by nighttime aircraft noise exposure, is a major health problem, affecting large parts of the population. This is the first study showing that noise annoyance is associated with increased levels of MR-proANP being a biomarker for the increased risk of incident cardiovascular events and death, providing further mechanistic insight to explain and support the increased cardiovascular risk observed in epidemiological noise studies. These results may contribute to a better understanding of pathophysiological mechanisms involved in the noise-annoyance-disease relationship, allowing to develop specific (pharmacological) interventions and preventive measures. Environmental noise exposure should be regarded as a manifest cardiovascular risk factor, which can hardly be modified by patients or physicians but rather by politicians and legislators, introducing noise limits that reduce the adverse cardiovascular effects of noise. Therefore, measures such as inclusion into the new European Society of Cardiology guidelines for cardiovascular prevention [42] should be implemented.
